# Regulation of proinflammatory genes by the circulating microRNA hsa-miR-939

**DOI:** 10.1038/srep30976

**Published:** 2016-08-08

**Authors:** Marguerite K. McDonald, Sujay Ramanathan, Andrew Touati, Yiqian Zhou, Rushi U. Thanawala, Guillermo M. Alexander, Ahmet Sacan, Seena K. Ajit

**Affiliations:** 1 Pharmacology & Physiology, Drexel University College of Medicine, 245 North 15th Street, Philadelphia, PA 19102, USA; 2 Gene Therapy Program, Perelman School of Medicine, University of Pennsylvania, Suite 2000, Translational Research Laboratories (TRL), 125 S. 31st Street, Philadelphia, PA 19104-3403, USA; 3 School of Biomedical Engineering, Science & Health Systems, Drexel University, 3141 Chestnut Street, Philadelphia, PA 19104, USA; 4 Neurology, Drexel University College of Medicine, 245 North 15th Street, Philadelphia, PA 19102, USA

## Abstract

Circulating microRNAs are beneficial biomarkers because of their stability and dysregulation in diseases. Here we sought to determine the role of miR-939, a miRNA downregulated in patients with complex regional pain syndrome (CRPS). Hsa-miR-939 is predicted to target several proinflammatory genes, including IL-6, VEGFA, TNFα, NFκB2, and nitric oxide synthase 2 (NOS2A). Binding of miR-939 to the 3′ untranslated region of these genes was confirmed by reporter assay. Overexpression of miR-939 *in vitro* resulted in reduction of IL-6, NOS2A and NFκB2 mRNAs, IL-6, VEGFA, and NOS2 proteins and NFκB activation. We observed a significant decrease in the NOS substrate l-arginine in plasma from CRPS patients, suggesting reduced miR-939 levels may contribute to an increase in endogenous NOS2A levels and NO, and thereby to pain and inflammation. Pathway analysis showed that miR-939 represents a critical regulatory node in a network of inflammatory mediators. Collectively, our data suggest that miR-939 may regulate multiple proinflammatory genes and that downregulation of miR-939 in CRPS patients may increase expression of these genes, resulting in amplification of the inflammatory pain signal transduction cascade. Circulating miRNAs may function as crucial signaling nodes, and small changes in miRNA levels may influence target gene expression and thus disease.

Distinct expression patterns of circulating microRNAs (miRNAs) have been associated with a wide range of diseases^1^. Widely recognized for their role as fine tuners of gene expression, miRNAs that mediate posttranscriptional regulation influence virtually all aspects of cellular processes^2^,^3^. These small noncoding RNAs regulate gene expression by binding predominantly to the 3′ untranslated region (3′UTR) of mRNAs by 6- to 8-basepair seed sequence complementarity. Upon binding, miRNAs can induce mRNA degradation or translational repression and thus negatively regulate the expression of target genes^2^,^3^.

Complex regional pain syndrome (CRPS) is a chronic neuropathic disorder involving sensory, motor, and autonomic dysregulation. Though the mechanisms underlying the development of pain are not fully understood, inflammation is known to play a crucial role in CRPS^4^,^5^,^6^. Studies investigating changes in inflammatory mediators in plasma, cerebrospinal fluid, and blisters from CRPS patients and healthy control subjects established that CRPS patients have significantly increased proinflammatory cytokines and reduced systemic levels of anti-inflammatory cytokines compared with controls^7^. In a previous study, we identified differential expression of 18 circulating miRNAs in whole blood from CRPS patients. Of the 18 differentially regulated miRNAs, miR-939 ranked first and showed a 4.3-fold downregulation (*p* value 6.0E-06) in CRPS patients^8^. Bioinformatic predictions showed that miR-939 can potentially target several mRNAs encoding various proinflammatory mediators, including interleukin-6 (IL-6), vascular endothelial growth factor (VEGFA), tumor necrosis factor α (TNFα), nitric oxide synthase 2 (NOS2A or iNOS), and nuclear factor-κB2 (NFκB2)^9^,^10^. Among these putative target genes, plasma levels of IL-6 and VEGF protein were significantly negatively correlated with miR-939 expression in patients with CRPS when compared to control^8^. This suggests that a reduction in miR-939 may contribute to an increase in the translation of these target mRNAs.

The classic inflammatory response occurring after injury includes secretion of proinflammatory cytokines. Since several of the predicted miR-939 target genes play a central role in regulation of the immune system^11^,^12^, we hypothesized that the downregulation of miR-939 may result in the upregulation of several mRNAs harboring miR-939 binding sites, known to regulate the inflammatory response in patients. Here, we have investigated the role of miR-939 in regulating the expression of inflammatory genes that may contribute to the disease etiology in CRPS and pain. While CRPS symptoms can be localized, elevations in inflammatory protein and decrease in miR-939 were observed systemically. Though miR-939 have been identified in primates, a rodent miR-939 homologue has not been reported to date. For these reasons, we chose human monocytic and endothelial cell lines, representing two cell types in constant contact with circulating molecules, for *in vitro* studies. Results from our *in vitro* studies and analyses of total RNA from whole blood and plasma from CRPS patients and controls suggest that downregulation of miR-939 in CRPS patients may increase the translation of proinflammatory target mRNAs.

## Results

### Confirmation of miR-939 binding to the 3′UTR of predicted targets

**Figure 1:**
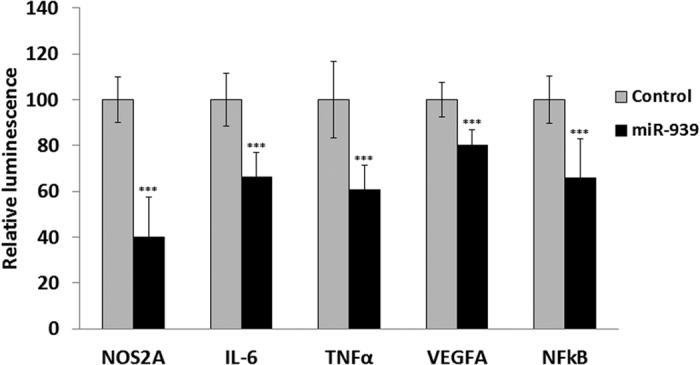
Luciferase assay to determine miR-939 binding to the 3′UTR of predicted mRNA targets. Plasmids with a target 3′UTR cloned downstream of the luciferase open-reading frame were cotransfected with plasmids encoding precursor miR-939 or control miRNA in HEK293 cells. Luciferase activity was measured 48 hours after transfection, and the data expressed as percentage of control. Firefly luciferase measurements normalized to Renilla was used as a transfection control. The average of 3 independent experiments is shown. Statistically significant difference from control was calculated using Student t-test ***p < 0.001.

We relied on multiple *in silico* prediction algorithms^9^,^10^ to identify putative inflammation and pain-related target genes for miR-939. The 3′UTRs of NOS2A, IL-6, TNFα, VEGFA, and NFκB2 harboring miR-939 binding sites were cloned downstream of the luciferase open-reading frame. HEK293 cells were transiently transfected with plasmids encoding the reporter 3′UTR constructs and either precursor miR-939 or a scrambled precursor miRNA control. Firefly luciferase measurements were normalized to Renilla as a transfection control. A significant reduction was observed in luciferase activity 48 hours after transfection with miR-939, (p < 0.001 compared to scrambled miRNA control) confirming the binding of miR-939 to the 3′UTRs of these predicted miR-939 targets (Fig. 1).

### Transcriptional regulation of target genes IL-6, TNFα, VEGFA, and NFκB2 by miR-939

In order to test the ability of miR-939 to modulate endogenous mRNAs involved in inflammation *in vitro*, we transfected human acute monocytic leukemia cells (THP-1) with miR-939 mimic or scrambled control miRNA. Since miRNAs can regulate gene expression by mRNA degradation or translational repression, we performed qPCR to measure endogenous levels of target mRNAs, 24 hours after transfection. In naïve THP-1 cells, a reduction in IL-6 mRNA levels were observed upon miR-939 transfection, while the other predicted target mRNA levels remained unchanged (see Supplementary Fig. S1), which suggested that miR-939 may reduce only a subset of mRNA through degradation. THP-1 cells are widely used to study inflammatory response because they can be stimulated with bacterial lipopolysaccharide (LPS) to generate proinflammatory cytokines. In order to assess the observable effects of miR-939 on induced levels of these target genes, otherwise normally found at very low levels, we stimulated the THP-1 cells with LPS. The time courses for mRNA induction after LPS treatment differed for the miR-939 target genes. In THP-1 cells, highest levels of induction for TNFα and VEGFA were observed at 1 h; NFκB2 transcripts in THP-1 cells reached the maximal level at 3 h post LPS stimulation; IL-6 increased gradually and peaked at 12 h (see Supplementary Fig. S2). Since NOS2A was not induced by LPS in THP-1 cells, we used HUVEC cells to investigate NOS2A transcript levels. NOS2A mRNA increase peaked at 1 h post LPS treatment (see Supplementary Fig. S3).

**Figure 2:**
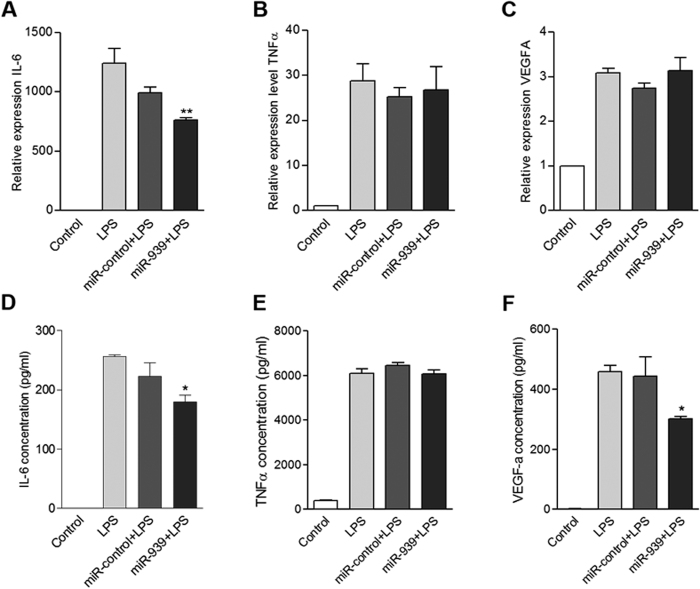
Expression levels of miR-939 target genes IL-6, TNFα and VEGFA in THP-1 cells transfected with miR-939 followed by 6 hours of stimulation with LPS. (**A–C**) Taqman analysis of endogenous levels of IL-6, TNFα and VEGFA, after LPS induction, showed that transfection with miR-939 reduced IL-6 transcripts compared to miR- control. (**D–F**) Overexpression of miR-939 decreased secreted IL-6, and VEGFA. ELISA using cell culture supernatants of THP-1 cells stimulated with LPS showed lower levels of these two proinflammatory mediators secreted by miR-939 transfected cells compared to control miRNA transfection. Significance was determined by one-way ANOVA with Dunnet’s post hoc test *p < 0.05, **p < 0.01.

We next determined the mRNA levels of TNFα, VEGFA and IL-6 in THP-1 cells stimulated with LPS following transfection with miR-939. Transfection of THP-1 cells with miR-939 significantly reduced the IL-6 transcripts in comparison to control. However, over expression of miR-939 did not lead to a significant downregulation of either TNFα or VEGFA mRNAs induced by LPS treatment (Fig. 2A–C, Supplementary Fig. S4). NFκB2 transcripts in THP-1 cells were significantly reduced by over expressing miR-939 at 3 h post LPS stimulation (see Supplementary Fig. S4). We also confirmed over expression of miR-939 in both THP-1 and HUVEC cells at 1, 3, 6 and 12 h post transfection (see Supplementary Fig. S5).

### miR-939 mediated changes in secreted IL-6, TNFα, and VEGFA proteins following LPS stimulation

Conditioned media from THP-1 cells transfected with miR-939 or control and stimulated with LPS were analyzed by ELISA for IL-6, TNFα, and VEGFA protein levels. The overexpression of miR-939 reduced the protein levels of IL-6 and VEGFA, but not TNFα being secreted into the media (Fig. 2D–F). Thus, over expression of miR-939 resulted in downregulation of both mRNA and protein levels for IL-6, and VEGFA protein but not its mRNA. TNFα mRNA and protein however were not significantly regulated by miR-939 (Fig. 2).

### Overexpression of miR-939 reduced NFκB activation following LPS stimulation

**Figure 3:**
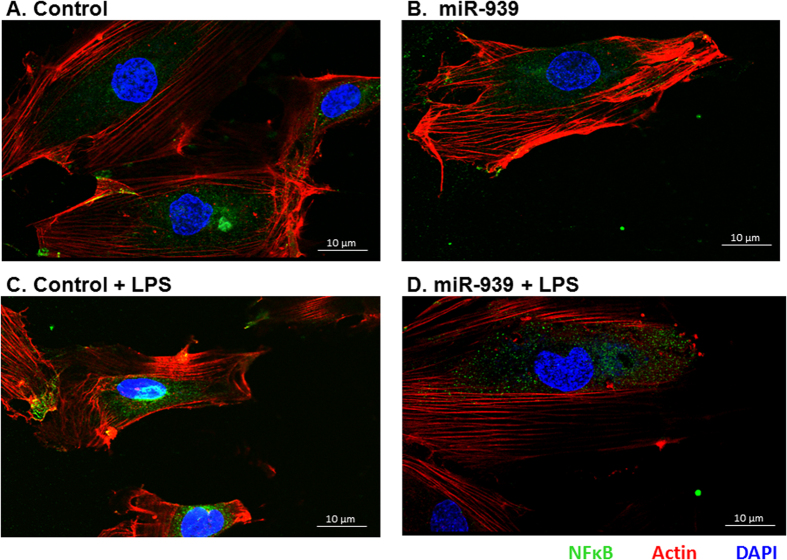
miR-939 reduced the translocation of functional NFκB to nucleus in response to LPS stimulation. HUVEC cells were transfected with miR-939 for 24 h, followed by LPS stimulation for 6 h and fixed. Immunostaining was performed with anti-NFκB antibody (green), Actin (red) and DAPI (blue). While LPS stimulation of HUVEC cells results in the translocation of NFκB to the nucleus (**C**) in comparison to untreated control (**A**), this is impaired in cells transfected with miR-939 (**D**), suggesting that a potential regulation of NFκB2 subunit by miR-939 can influence the dimerization and function of NFκB.

**Figure 4:**
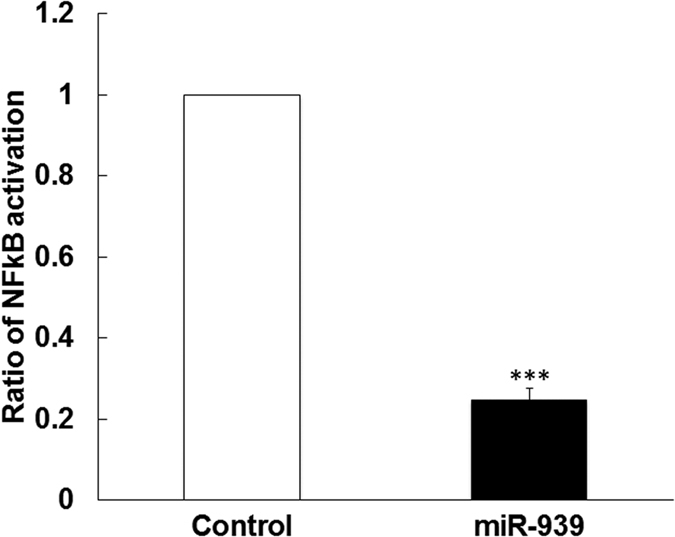
miR-939 reduced the activation of NFκB in response to LPS. THP-1XBlue cells, which have an inducible, chromosomally integrated secreted alkaline phosphatase (SEAP) gene downstream of the NFκB promoter, were transfected with miR-939 and then stimulated with LPS for 1 hour. The presence of high levels of miR-939 reduced the amount of SEAP detected in the media indicating less NFκB activity. Statistical significance was calculated using Student t-test ***p < 0.001.

Similarly, in order to investigate the role of miR-939 in regulating NFκB activity, we overexpressed miR-939 and stimulated either HUVEC cells or THP-1 cells with LPS. Treatment of HUVEC cells with LPS is known to aid the translocation of NFκB into the nucleus, resulting in a higher NFκB activity and subsequent upregulation of adhesion molecules^13^. We performed immunostaining in HUVEC cells transfected with miR-939 or control miRNA which show that NFκB translocates to the nucleus 6 h after LPS stimulation (Fig. 3C), and this translocation was impaired in the presence of miR-939 (Fig. 3D). To determine if the overexpression of miR-939 can modulate NFκB function, we performed a reporter assay using THP-1XBlue cells, which have an inducible, chromosomally integrated secreted alkaline phosphatase (SEAP) gene downstream of the NFκB promoter. In this assay, transfection with miR-939 reduced the activation of NFκB in response to LPS, indicating a functional role for miR-939 (Fig. 4). Taken together, these data suggest that miR-939 can significantly reduce the nuclear translocation and successive activation of NFκB in response to LPS.

### miR-939 can regulate NOS2A mRNA and protein expression *in vitro*

**Figure 5:**
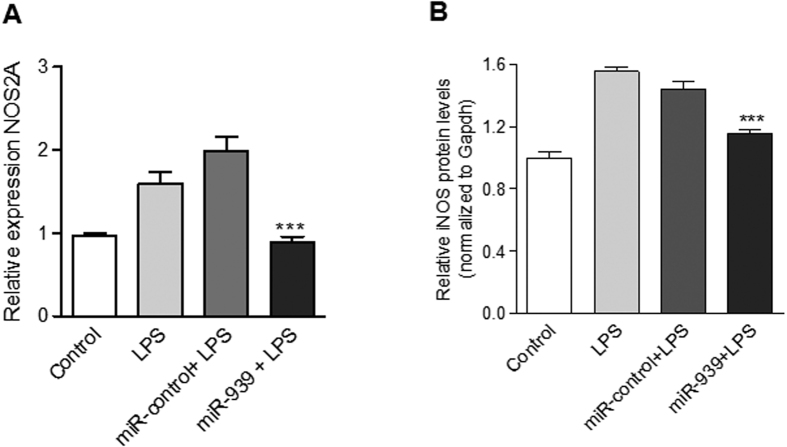
Overexpression of miR-939 decreases NOS2A mRNA and protein levels in HUVEC cells. (**A**) Taqman analysis of endogenous levels of NOS2A mRNA in HUVEC cells determined 24 hours after transfection with miR-939, and LPS treatment for 1 h. GAPDH was used as a normalizer. (**B**) ELISA for NOS2 in HUVEC cells transfected with miR-939 followed by LPS treatment for 6 h, show that NOS2 levels were lower after miR-939 transfection compared to control. Values are the mean of three experiments. Significance was determined by one-way ANOVA with Dunnet’s post hoc test ***p < 0.001.

We had observed that miR-939 can decrease endogenous NOS2A mRNA in HUVEC cells without LPS stimulation, but not in THP-1 cells (see Supplementary Fig. S1). As NOS2 is known to play a key role in pain and inflammation, we sought to determine if miR-939 could modulate its mRNA and protein levels, *in vitro*. We observed that LPS stimulation led to an increase in NOS2A mRNA levels in HUVEC cells, which was significantly attenuated upon transfection with miR-939 (Fig. 5A). To further investigate if miR-939 can regulate the cellular NOS2 protein levels, we analyzed the cell lysates of miR-939 and control miRNA transfected HUVEC cells following LPS treatment, using a cell-based ELISA, which shows that miR-939 can lower the LPS-induced NOS2 levels, when compared with cells transfected with control miRNA (Fig. 5B).

### Plasma levels of l-arginine in CRPS patients as a measure of NOS2 activity

**Figure 6:**
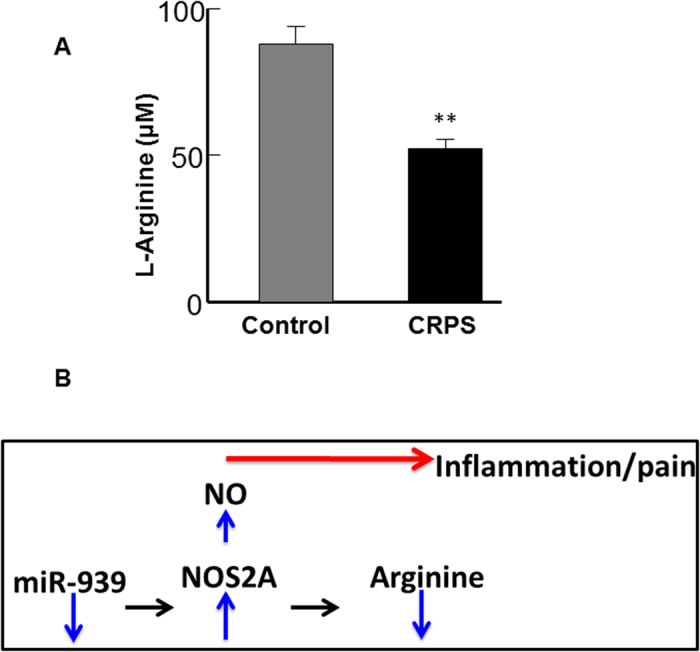
Arginine levels in plasma from CRPS patients and control individuals. The NOS2 substrate, l-arginine, was downregulated in plasma from CRPS patients (n = 41) compared to control individuals (n = 20). Statistical significance was calculated using Student t-test **p < 0.01. Downregulation of miR-939 may be linked to inflammation in CRPS patients (diagram). Increase in NOS2 protein, with the consequent decrease in its substrate, l-arginine, may result in enhanced NO levels, leading to pain and inflammation.

In concert with the above findings that show the *in vitro* regulation of proinflammatory target genes by miR-939, in our previous study, we had observed that plasma levels of IL-6, VEGFA, and TNFα proteins were upregulated in all, or in a subset of CRPS patients stratified based on miRNA profile^8^. However, we could not assess the protein levels of the other two target genes, NFκB2 and NOS2 in patient samples. Therefore, we employed an indirect measure to assess changes in NOS2 activity in the plasma. l-arginine is the substrate for NOS, resulting in the production of NO and l-citrulline. Concurrently, when we measured the arginine levels in plasma obtained from 41 CRPS patients and in 20 control samples that were used in the miRNA profiling study^8^, we found that patients with CRPS had significantly decreased arginine levels (Fig. 6A), possibly indicating increased levels or activity of NOS2. Based on these observations, we hypothesize that the downregulation of miR-939 may contribute to an overall increase in NOS2 activity, which would result in lower arginine levels and increased NO production, leading to pain and inflammation (Fig. 6B).

### Expression of miR-939 target genes in whole blood from CRPS patients

**Figure 7:**
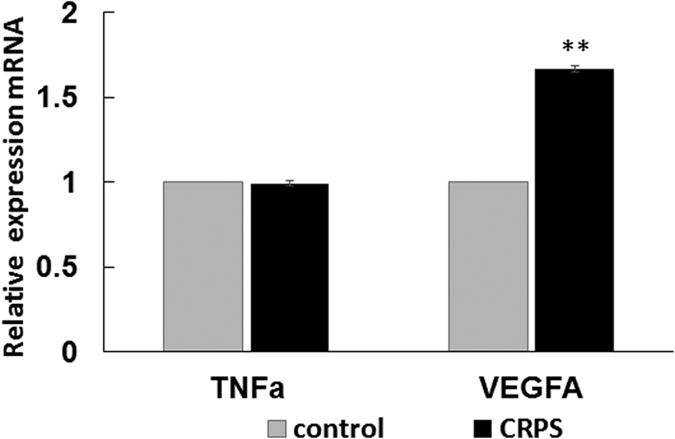
Relative expressions of VEGFA and TNFα mRNA in whole blood from CRPS patients and control individuals. Taqman analysis of target mRNAs of miR-939 that were consistently detectable in whole blood, showed a significant increase of VEGFA mRNA in patients with CRPS compared to control samples. TNFα mRNA levels were not significant between CRPS and control samples. GAPDH was used as the normalizer. Data represent mean ± SEM CRPS (n = 37) and control (n = 18). Statistical significance was calculated using Student t-test **p < 0.01.

The variable regulation exerted by miR-939 on its target genes observed *in vitro* led us to investigate the expression levels of target genes in whole blood from CRPS patients. Using the total RNA isolated for our initial miRNA profiling^8^, we performed qPCR analysis for genes that could be reliably detected in whole blood. IL-6 and NOS2A mRNA were undetectable by qPCR. We observed a significant increase in VEGFA mRNA (Fig. 7), but not TNFα mRNA, in the whole blood of patients with CRPS compared to control. Thus, while both the *in vitro* (Fig. 2F) and whole blood analysis^8^ showed an inverse correlation between miR-939 and VEGFA protein, changes in the VEGFA mRNA was only significant in blood (Fig. 7) and not in our *in vitro* studies (Fig. 2C).

### miR-939 represents a critical regulatory node in a network of inflammatory mediators

**Figure 8:**
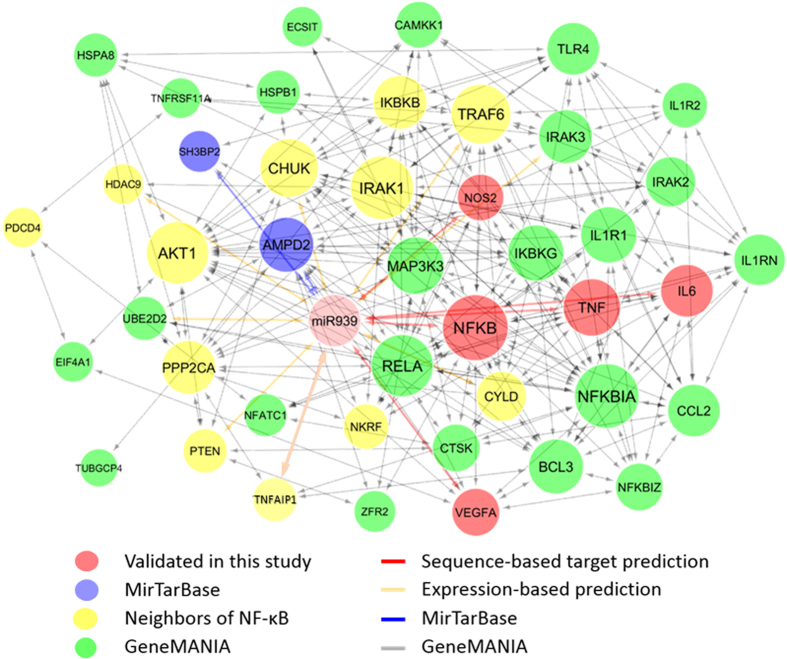
The inflammatory gene network representing miR-939 involvement. A seed list of genes constructed from validated targets of miR-939 and interaction neighbors of NFκB, was enriched with functionally associated genes, using GeneMANIA. The nodes are colored according to the source of information. The connections between genes indicate a functional association; those between miR-939 and genes indicate validated or computationally predicted targets (Arrowheads suggest association, but do not indicate directionality in the interaction).

In order to investigate how miR-939 participates in the inflammatory gene interaction network, and its role in mediating inflammation and pain, we constructed a network from the validated and predicted targets of miR-939 and the genes that have a known functional association with these targets. The resulting set of 42 genes is shown in Fig. 8, with connections between genes indicating a known functional association available in GeneMANIA (green). In addition to the computationally predicted targets from TargetScan, we found coexpression-based targets of miR-939 (HDAC9, PTEN, TRAF6, IRAK3, CHUK, UBE2D2, CYLD) from five paired miRNA–mRNA expression datasets available from Gene Expression Omnibus (GEO datasets—GSE19536, GSE35602, GSE40355, GSE21032, GSE51993)^14^—using a regression-based method^15^. These coexpression-based targets are likely the result of indirect regulation by one or more direct targets of miR-939.

## Discussion

A loss in the buffering role of one miRNA can influence the expression of its mRNA targets, resulting in a drift from normal cellular function^16^,^17^. Emerging evidence indicates that some miRNAs may exert their full impact on their target genes only under certain conditions, such as specific signaling cues, stress, or developmental stages^3^. Cellular context may also influence miRNA targeting^18^. Hence, THP-1 and HUVEC, representing two cell types in constant contact with circulating miRNAs, were chosen for *in vitro* studies. Our *in vitro* data indicate that miR-939-mediated gene regulation may be occurring predominantly by translational repression at the time points we investigated. The miR-939-induced mRNA degradation that we observed, differed between the two cell types, with decreases observed in endogenous IL-6 and NOS2A mRNA in THP-1 and HUVEC cells, respectively. This cell type–dependent effect exerted by miR-939 suggests that cellular context may play a significant role in the mode of gene regulation. Occlusion of potential miRNA binding sites on mRNA by RNA binding proteins (RBPs), or lack of access due to a secondary structure of mRNA, could also contribute to alterations in miRNA binding^2^. To further investigate whether mRNA induction could influence miRNA binding, we stimulated THP-1 cells with LPS, 24 hours after miR-939 transfection. While LPS stimulation significantly induced IL-6, TNFα, and VEGFA, only IL-6 levels decreased in the presence of miR-939. This finding suggests that the inhibitory effect of miR-939 on IL-6 mRNA levels remained similar under rapid upregulation of target mRNAs in THP-1 cells. Although interference with translation initiation is emerging as the target of miRNA function^3^, numerous RBPs^19^ and AU-rich elements (AREs)^20^ can also regulate the stability of mRNAs. Additional studies are needed to elucidate whether conformational changes or RBPs play a role in regulating miR-939 function.

We extended our studies to RNA obtained from whole blood of CRPS patients who participated in our previous miRNA profiling study^8^. We focused on target genes reliably detected by qPCR in whole blood and found a significant increase in VEGFA mRNA, but not for TNFα mRNA, in samples from patients with CRPS compared with control samples. Previous studies, including ours, have reported elevation of VEGFA, TNFα, and IL-6 in plasma^8^,^21^, cerebrospinal fluid, blister fluids, and skin biopsies from CRPS patients^7^. Although downregulation of miR-939 alone cannot account for these changes, it is reasonable to assume that miR-939 could repress the translation of inflammatory proteins, instead of degrading the transcript. Our *in vitro* studies showed that the secretion of IL-6, and VEGFA proteins in culture media from cells transfected with miR-939 was downregulated, while TNFα was not. Although the luciferase assay performed in HEK293 cells confirmed the binding of miR-939 to the 3′UTR of TNFα, and a subset of patients had elevated TNFα in plasma, our studies in HUVEC and THP-1 cells did not show significant alteration in TNFα mRNA or protein upon miR-939 overexpression. Additional studies using primary immune cells are needed to determine if the lack of efficacy of miR-939 in regulating TNFα is a cell-type specific effect.

Transcription factor NFκB plays a central role in inflammation, and is a master regulator of both the innate and adaptive immune systems^11^. The pleiotropic nature of the NFκB signaling allows for temporal regulation of inflammation. Proinflammatory gene transcription ensues in the early phase of immune response, which then transitions to anti-inflammatory gene transcription later in the progression of inflammation^22^. NFκB has been proposed to be a central mediator in both the initiation and progression of CRPS^23^. NFκB is involved in the development of allodynia in an animal model that mimics the symptoms of CRPS-1, a subtype of the disease that occurs after an injury that did not directly damage the nerves^5^,^24^. A number of miRNAs have been implicated in the regulation of NFκB signaling^11^,^25^ including miR-939. A noncanonical mechanism of action for miR-939 and NFκB was shown in a recent study^26^. miR-939 physically interacted with NFκB and this was mediated by the cis-elements present within the sequences of miR-939 for the p50 subunit of NFκB. It was proposed that miR-939 can act as endogenous decoy molecules for NFκB^26^. Our *in vitro* studies and pathway analysis indicate that the regulation of NFκB expression by miR-939 can directly and indirectly impact several proinflammatory mediators.

Members of the NOS family of enzymes, including endothelial, neuronal, and inducible NOS, catalyze the production of NO from l-arginine. NOS2A (inducible NOS) is expressed primarily in inflammatory cells and is activated by proinflammatory cytokines, resulting in high NO levels^27^. NO may participate in or induce cell injury, and this could be the primary cause of chronic pain^28^. Increased NO production has been observed in interferon-γ-stimulated peripheral blood monocytes obtained from CRPS patients^29^. A recent study investigating regulation of NOS2A expression demonstrated a functional role for miR-939; NOS2 3′UTR conferred significant post-transcriptional blockade of luciferase activity in human A549, HCT8, and HeLa cells^30^. They demonstrated that the transfection of miR-939 into primary human hepatocytes significantly inhibited cytokine-induced NO synthesis in a dose-dependent manner. Our studies showed that miR-939 decreased NOS2 protein levels in HUVEC cells. Thus, the functional repression we observed indicates that miR-939 binding to the 3′UTR of NOS2A is consistent across a multitude of cell lines. A significant decrease in plasma arginine levels in CRPS patients also corroborates with the above findings, and suggests that the downregulation of miR-939 may contribute to an increase in NOS2A and NO production, leading to pain and inflammation.

We investigated the role of miR-939 in mediating inflammation and pain, using a gene interaction network. In addition to validated and predicted targets of miR-939, genes with known functional associations with miR-939 targets were included in the analysis. The resulting densely connected network shows the complexity of the interactions among the inflammatory genes. By targeting multiple genes in this network, miR-939 may achieve a rapid signaling cascade, affecting many of the downstream proteins and phenotypes. While the signaling cascades involving the inflammatory genes are the subject of ongoing research, we believe that network enrichment is useful to identify key players involved in the molecular mechanisms of inflammatory pain. Collectively these data suggest that miR-939 represents a critical node for interaction with parallel signaling pathways; in its role as a signaling mediator, miR-939 may integrate diverse stimuli to achieve cell- and stimulus-specific responses. In addition to directly regulating multiple proinflammatory mRNAs, the upregulation of some proteins such as IL-6 may have a cascading effect in their role as positive regulators for other miR-939 target genes. Thus downregulation of miR-939 in CRPS patients may lead to a concomitant increase in target gene expression, promoting inflammation and pain.

miR-939 has been functionally associated with the replication of hepatitis B virus^31^. In systemic lupus erythematosus, an autoimmune inflammatory disease, expression of miR-939 was elevated in a specific subset of patients with different autoantibody specificities^32^. Our studies indicate that miR-939 function is closely integrated with the inflammatory gene regulatory network. The key role of the neuroimmune interface in persistent pain is well known^33^, and circulating miRNAs may contribute to chronic pain by linking various components of neuroimmune signaling. miRNA combinatorial regulation has been proposed as an attractive regulatory strategy in modulating pathways and cellular homeostasis^34^. Because our studies indicate a role for miR-939 in proinflammatory signal amplification, increasing miR-939 levels should restore homeostasis by decreasing inflammatory protein synthesis in CRPS patients.

The source of miR-939 in circulation is currently unknown. Extracellular miRNAs may be present in circulation bound to argonaute proteins, or they may be transported in exosomes^35^. Exosomal composition is unique to disease and the specificity of molecular signature observed in biomolecular cargo can be explored for its biomarker utility. Our previous study investigating the exosomal fraction of blood from CRPS patients showed a significant upregulation of miR-939, though it was downregulated in whole blood^36^. This negative correlation in expression of miR-939 between whole blood and exosomes leads us to propose a protective role for exosomes in CRPS. The presence of increased amounts of miR-939, an miRNA that can potentially modulate proinflammatory mRNAs in recipient cells, may indicate a systemic attempt to resolve the chronic condition via exosome-mediated transport of miRNAs^36^. Exosomal biology is an emerging area of research and the mechanisms whereby miRNAs are sorted to exosomes and the significance of miRNA transfer to acceptor cells are still being elucidated. A recent study showed that miRNA availability for exosomal secretion is controlled, at least in part, by the cellular levels of their targeted transcripts^37^. Thus changes in transcriptome in response to cellular activation may modulate miRNA sorting to the exosomes. This may imply that exosomal miRNA secretion is a mechanism whereby cells rapidly dispose miRNAs in excess of their targets to adjust miRNA:mRNA homeostasis. Additional studies are needed to confirm the regulatory role of miR-939 transported in exosomes and to determine whether inflammation can upregulate miR-939 packaging into exosomes. We postulate that uptake of exosomes enriched in miR-939 can decrease proinflammatory gene expression in recipient cells. Future studies will also focus on investigating aberrations in exosomal uptake in immune cells from CRPS patients. This will be key in understanding if impaired uptake and thus signaling is an underlying disease mechanism in CRPS. From a biomarker perspective, most of the studies reporting alterations in circulating miRNAs for neuropathic conditions had limited cohort size^35^,^38^. Validating the findings in bodily fluids or purified exosomes from a larger number of patients is required to determine whether miRNA could be a useful biomarker for both patient stratification and predicting treatment response. Retrospective analysis of banked samples can be pursued because exosomes are stable and thus can be purified from stored serum samples.

Collectively, our data suggest that miR-939 may regulate multiple proinflammatory genes and that downregulation of miR-939 in CRPS patients may contribute to an increase in expression of these target genes. A relatively small change in miR-939 in CRPS patients has the potential to modulate and amplify the proinflammatory signal transduction cascade that may contribute to chronic inflammation and pain. Thus, understanding the mechanistic significance of miRNAs in relation to their interaction with other neuronal and immune components modulated under chronic pain states, may offer insights into the signal transduction processes that underlie inflammation and pain.

## Methods

### Study approval

All subjects were enrolled after giving informed consent as approved by the Drexel University College of Medicine Institutional Review Board. The methods were carried out in accordance with the approved guidelines. All experimental protocols were approved by Drexel University College of Medicine Institutional Review Board.

### Cell culture

HEK293 cells (American Type Culture Collection [ATCC]) was maintained in Dulbecco’s Modified Eagle’s Medium (DMEM) supplemented with 10% fetal bovine serum at 37 °C in 5% CO_2_. Human monocytic THP-1 cells (from ATCC) or THP1-XBlue (InvivoGen; San Diego, CA) cells were maintained in ATCCformulated RPMI1640 medium containing 10% fetal bovine serum (FBS). Human umbilical vein endothelial cells (HUVECs) were maintained in Endothelial Growth Medium (EGM2 bullet kit) from Lonza (Allendale, NJ).

### Luciferase reporter assay

The 3′UTR luciferase reporter constructs for IL-6 (NM_000600.2), VEGFA (NM_001033756.1), and NFκB2 (NM_001077494.1) were purchased from GeneCopoeia (Rockville, MD), and those for TNFα (NM_000594) and NOS2A (NM_000625.4) were purchased from OriGene (Rockville, MD). HEK293 cells were cotransfected with precursor miRNA expression clone for human miR-939 (HmiR0533-MR04) or with precursor miRNA scrambled control (GeneCopoeia) and luciferase reporter plasmid containing the 3′UTR of genes of interest using Lipofectamine 2000 (Life Technologies, Carlsbad, CA) for 48 hours. The Luc-Pair miR Luciferase assay kit (GeneCopoeia) was used to measure firefly and Renilla luciferase activity according to the manufacturer’s instructions. Firefly luciferase measurements normalized to Renilla was used as a transfection control. The data expressed as percentage of control is the average of 3 independent experiments.

### miR-939 overexpression in HUVEC and THP-1 cells

Transfections were performed following the manufacturer’s protocol for RNAiMax transfection reagent using either miR-939-5p mimic (MC 12517) or negative control mimic (4464058) (Life Technologies) with the following modifications. For each well of a 6-well plate, 7.5 μl RNAiMax reagent was diluted in 150 μl of serum-free media, and 30 pmol of miR-939 or control mimic was diluted in 150 μl of serum-free media individually. The dilutions were combined and incubated at room temperature for 15 minutes. This transfection complex (300 μl) was added to 0.5 × 10^6^ cells/well in 1.7 ml serum containing media in 6-well plates and incubated for 6 hours at 37 °C, after which the media was changed. After 24 hours, cells were treated with 1 μg/ml lipopolysaccharide (LPS) in complete culture media. Cells were collected by centrifugation at 135 × *g* for 5 minutes at 4 °C and the conditioned media was stored at 4 °C. The cell pellet was washed with 1× phosphate-buffered saline and resuspended in either RNA lysis buffer (mirVana kit; Life Technologies) containing 0.5 U/μl RNAsin Plus (Promega; Madison, WI) or radioimmunoprecipitation assay (RIPA) buffer containing protease inhibitor cocktail (Thermo Scientific; Waltham, MA).

### cDNA synthesis and qPCR for mRNAs and miR-939

mRNA was isolated using the miRVana kit (Life technologies). The Maxima cDNA synthesis kit (Thermo Scientific) was used to generate cDNA and 2 μl cDNA was used for Taqman based quantitative real time mRNA analysis containing 10 μl Taqman Fast Universal polymerase chain reaction (PCR) master mix (2×) no AmpErase UNG (Life Technologies), 1 μl Taqman primer probe (20×), in up to 20 μl nuclease-free water. GAPDH was used as the normalizer and one way ANOVA was used to perform statistical analysis. Assay IDs were as follows: Hs01075529_m1 [NOS2], Hs01113624_g1 [TNFα], Hs00900055_m1 [VEGFA], Hs00985639_m1 [IL-6], Hs01028901_g1 [NFκB2] (Applied Biosystems, Carlsbad, CA). For miR-939 qPCR, cDNA synthesis and detection was performed using TaqMan microRNA assay (Assay ID 002182, Applied Biosystems). U6 snRNA was used for normalization (Assay ID 001973).

### Enzyme-linked immunosorbent assay (ELISA)

Supernatants collected after miR-939 or control miRNA transfections were used to perform ELISA for secreted proteins IL-6, TNFα, and VEGFA according to the manufacturer’s protocol (R&D Systems; Minneapolis, MN). Cell based ELISA for human iNOS activity was performed as per manufacturer’s instruction (R&D systems). Briefly, HUVEC cells (1 × 10^4^ cells/well) were grown in a 96 well clear bottom black fluorescence plate and transfected with miRNA. After 24 hours, cells were treated with LPS for 6 hours. The cells were then fixed in 4% paraformaldehyde for 20 min, washed and incubated in blocking buffer for 1 hour followed by primary antibody mixture (anti-iNOS and anti-GAPDH) overnight at 4 °C. The cells were washed and incubated with HRP- and AP-conjugated secondary antibodies for 2 h followed by further washing and incubation with the respective fluorescence substrates for 20 min. Fluorescence was measured using a Wallac Victor2 fluorescence plate reader (Perkin Elmer) with excitation at 540 nm and emission at 600 nm for total iNOS levels, and excitation at 360 nm and emission at 450 nm for total GAPDH in the cells. Relative iNOS expression was calculated from fluorescence units of iNOS normalized to GAPDH for each sample.

### NFκB Immunocytochemistry

HUVEC cells (1 × 10^4^ cells) were cultured on poly-lysine treated cover slips in EGM2 bullet kit (Lonza) for 16 hours and transfected with the miRNA. After 24 hours, cells were treated with LPS for 6 hours. The cells were then fixed in 4% paraformaldehyde for 5 min at 4 °C, permeabilized with 20% v/v Methanol for 5 min at 4 °C, and blocked with 3% normal goat serum (NGS) in 1X PBS containing 0.1% Triton-X-100 for 30 min. The cells were incubated overnight at 4 °C with rabbit anti-NFκB (ab131539, Abcam) in PBS containing 3% NGS and 0.2% Triton-X-100, washed thrice with PBS and blocked again with 10% NGS and washed thrice before staining with Alexafluor 488-conjugated anti-rabbit IgG antibody (A21206, Molecular probes), Texas Red^®^-X Phalloidin (T7471, Molecular probes) and DAPI for 30 min. The cells were washed twice with PBS and images were acquired using the Olympus FV1000 confocal microscope.

### NFκB reporter assay

THP1-XBlue cells maintained in complete media were seeded into a 96-well plate after transfection with RNAiMax with or without miR-939. Four hours after transfection, cells were stimulated with 1 μg/ml LPS and media was collected 4 hours after treatment. QUANTI-Blue assay was performed using QUANTI-Blue media prepared as described by the manufacturer (InvivoGen). To 150 μl QUANTI-Blue media, 50 μl conditioned media was added and incubated at 37 °C for 24 hours. Plates were read at 650 nm (Spectramax Plus, Molecular Devices; Sunnyvale, CA).

### Arginine measurement

Plasma levels of arginine were determined by high-performance liquid chromatography with fluorimetric detection as described previously^39^. The fluorescent amino acid derivative was detected using a Gilson (Middleton, WI) model 121 fluorometer.

### Statistical analysis

Data are presented as mean ± the standard error of the mean from three or more independent experiments. Student *t*-test was used for determining the statistical significance. Treatment effects were analyzed with a one way analysis of variance (ANOVA). Pairwise comparisons between means were tested using the *post hoc* Dunnet method. Error probabilities of *p* < 0.05 were considered statistically significant.

### Pathway analysis

A network was constructed from the validated and predicted targets of miR-939 and the genes that have known functional associations with these targets. The validated targets of miR-939 included the ones validated in this study (TNFα, IL-6, NOS2, VEGFA, NFκB2) and those available from the MirTarBase database (SH3BP2 and AMPD2)^40^. Because of the central role played by NFκB in inflammatory response, the interaction neighbors of NFκB (PPP2CA, TNFAIP1, TRAF6, IRAK1, NKRF, HDAC9, AKT1, PDCD4, CYLD, PTEN, CHUK, and IKBKB) were also included^41^. The list of miR-939 targets and the NFκB interaction neighbors was then enriched with their own neighbors using GeneMANIA^42^.

## Additional Information

**How to cite this article**: McDonald, M. K. *et al.* Regulation of proinflammatory genes by the circulating microRNA hsa-miR-939. *Sci. Rep.* **6**, 30976; doi: 10.1038/srep30976 (2016).

## Supplementary Material

Supplementary Information

## References

[b1] De Guire V. *et al.* Circulating miRNAs as sensitive and specific biomarkers for the diagnosis and monitoring of human diseases: promises and challenges. Clin Biochem 46, 846–860, doi: 10.1016/j.clinbiochem.2013.03.015 (2013). 23562576

[b2] Bartel D. P. MicroRNAs: target recognition and regulatory functions. Cell 136, 215–233, doi: S0092-8674(09)00008-7 (2009). 19167326 10.1016/j.cell.2009.01.002 PMC3794896

[b3] Wilczynska A. & Bushell M. The complexity of miRNA-mediated repression. Cell death and differentiation 22, 22–33, doi: 10.1038/cdd.2014.112 (2015). 25190144 PMC4262769

[b4] Schwartzman R. J., Erwin K. L. & Alexander G. M. The natural history of complex regional pain syndrome. The Clinical journal of pain 25, 273–280, doi: 10.1097/AJP.0b013e31818ecea5 (2009). 19590474

[b5] Bruehl S. An update on the pathophysiology of complex regional pain syndrome. Anesthesiology 113, 713–725, doi: 10.1097/ALN.0b013e3181e3db38 (2010). 20693883

[b6] Birklein F. & Schlereth T. Complex regional pain syndrome-significant progress in understanding. Pain 156 Suppl 1, S94–s103, doi: 10.1097/01.j.pain.0000460344.54470.20 (2015). 25789441

[b7] Parkitny L. *et al.* Inflammation in complex regional pain syndrome: A systematic review and meta-analysis. Neurology 80, 106–117, doi: 10.1212/WNL.0b013e31827b1aa1 (2013). 23267031 PMC3589200

[b8] Orlova I. A. *et al.* MicroRNA modulation in complex regional pain syndrome. J Transl Med 9, 195, doi: 1479-5876-9-195 (2011). 22074333 10.1186/1479-5876-9-195 PMC3228853

[b9] Grimson A. *et al.* MicroRNA targeting specificity in mammals: determinants beyond seed pairing. Molecular cell 27, 91–105, doi: 10.1016/j.molcel.2007.06.017 (2007). 17612493 PMC3800283

[b10] Liu B., Li J. & Cairns M. J. Identifying miRNAs, targets and functions. Briefings in Bioinformatics 15, 1–19, doi: 10.1093/bib/bbs075 (2014). 23175680 PMC3896928

[b11] Boldin M. P. & Baltimore D. MicroRNAs, new effectors and regulators of NF-kappaB. Immunological reviews 246, 205–220, doi: 10.1111/j.1600-065X.2011.01089.x (2012). 22435557

[b12] Ji R.-R., Xu Z.-Z. & Gao Y.-J. Emerging targets in neuroinflammation-driven chronic pain. Nat Rev Drug Discov 13, 533–548, doi: 10.1038/nrd4334 (2014). 24948120 PMC4228377

[b13] Jersmann H. P., Hii C. S., Ferrante J. V. & Ferrante A. Bacterial lipopolysaccharide and tumor necrosis factor alpha synergistically increase expression of human endothelial adhesion molecules through activation of NF-kappaB and p38 mitogen-activated protein kinase signaling pathways. Infection and immunity 69, 1273–1279, doi: 10.1128/iai.69.3.1273-1279.2001 (2001). 11179288 PMC98017

[b14] Edgar R., Domrachev M. & Lash A. E. Gene Expression Omnibus: NCBI gene expression and hybridization array data repository. Nucleic acids research 30, 207–210 (2002). 11752295 10.1093/nar/30.1.207 PMC99122

[b15] Zhou Y., Qureshi R. & Sacan A. Data simulation and regulatory network reconstruction from time-series microarray data using stepwise multiple linear regression. Netw Model Anal Health Inform Bioinforma 1, 3–17, doi: 10.1007/s13721-012-0008-4 (2012).

[b16] Emde A. & Hornstein E. miRNAs at the interface of cellular stress and disease. Embo j 33, 1428–1437, doi: 10.15252/embj.201488142 (2014). 24867813 PMC4194087

[b17] Mendell J. T. & Olson E. N. MicroRNAs in stress signaling and human disease. Cell 148, 1172–1187, doi: 10.1016/j.cell.2012.02.005 (2012). 22424228 PMC3308137

[b18] Nam J. W. *et al.* Global analyses of the effect of different cellular contexts on microRNA targeting. Molecular cell 53, 1031–1043, doi: 10.1016/j.molcel.2014.02.013 (2014). 24631284 PMC4062300

[b19] Jiang P. & Coller H. Functional interactions between microRNAs and RNA binding proteins. MicroRNA (Shariqah, United Arab Emirates) 1, 70–79 (2012). 10.2174/2211536611201010070 PMC5123774 25048093

[b20] Palanisamy V., Jakymiw A., Van Tubergen E. A., D’Silva N. J. & Kirkwood K. L. Control of cytokine mRNA expression by RNA-binding proteins and microRNAs. Journal of dental research 91, 651–658, doi: 10.1177/0022034512437372 (2012). 22302144 PMC3383846

[b21] Alexander G. M., Peterlin B. L., Perreault M. J., Grothusen J. R. & Schwartzman R. J. Changes in plasma cytokines and their soluble receptors in complex regional pain syndrome. The journal of pain : official journal of the American Pain Society 13, 10–20, doi: 10.1016/j.jpain.2011.10.003 (2012). 22172450

[b22] Lawrence T. The nuclear factor NF-kappaB pathway in inflammation. Cold Spring Harbor perspectives in biology 1, a001651, doi: 10.1101/cshperspect.a001651 (2009). 20457564 PMC2882124

[b23] Hettne K. M. *et al.* Applied information retrieval and multidisciplinary research: new mechanistic hypotheses in complex regional pain syndrome. J Biomed Discov Collab 2, 2, doi: 10.1186/1747-5333-2-2 (2007). 17480215 PMC1871567

[b24] de Mos M. *et al.* Role of NFkappaB in an animal model of complex regional pain syndrome-type I (CRPS-I). The journal of pain: official journal of the American Pain Society 10, 1161–1169, doi: S1526-5900(09)00504-5 (2009). 19878863 10.1016/j.jpain.2009.04.012 PMC4531089

[b25] Cheng H. S., Njock M. S., Khyzha N., Dang L. T. & Fish J. E. Noncoding RNAs regulate NF-kappaB signaling to modulate blood vessel inflammation. Front Genet 5, 422, doi: 10.3389/fgene.2014.00422 (2014). 25540650 PMC4261819

[b26] Cui C., Yu J., Huang S., Zhu H. & Huang Z. Transcriptional regulation of gene expression by microRNAs as endogenous decoys of transcription factors. Cellular physiology and biochemistry : international journal of experimental cellular physiology, biochemistry, and pharmacology 33, 1698–1714, doi: 10.1159/000362952 (2014). 24923223

[b27] Benarroch E. E. Nitric oxide: A pleiotropic signal in the nervous system. Neurology 77, 1568–1576, doi: 10.1212/WNL.0b013e318233b3e4 (2011). 22006889

[b28] Hancock C. M. & Riegger-Krugh C. Modulation of pain in osteoarthritis: the role of nitric oxide. The Clinical journal of pain 24, 353–365, doi: 10.1097/AJP.0b013e31815e5418 (2008). 18427234

[b29] Hartrick C. T. Increased production of nitric oxide stimulated by interferon-gamma from peripheral blood monocytes in patients with complex regional pain syndrome. Neuroscience letters 323, 75–77 (2002). 11911993 10.1016/s0304-3940(02)00112-x

[b30] Guo Z. *et al.* miRNA-939 regulates human inducible nitric oxide synthase posttranscriptional gene expression in human hepatocytes. Proceedings of the National Academy of Sciences of the United States of America 109, 5826–5831, doi: 10.1073/pnas.1118118109 (2012). 22451906 PMC3326458

[b31] Zhang X. *et al.* Plasma microRNA profile as a predictor of early virological response to interferon treatment in chronic hepatitis B patients. Antiviral therapy 17, 1243–1253, doi: 10.3851/imp2401 (2012). 22997154

[b32] Rai R., Chauhan S. K., Singh V. V., Rai M. & Rai G. Heat shock protein 27 and its regulatory molecules express differentially in SLE patients with distinct autoantibody profiles. Immunology letters 164, 25–32, doi: 10.1016/j.imlet.2015.01.007 (2015). 25655337

[b33] Grace P. M., Hutchinson M. R., Maier S. F. & Watkins L. R. Pathological pain and the neuroimmune interface. Nat Rev Immunol 14, 217–231, doi: 10.1038/nri3621 (2014). 24577438 PMC5525062

[b34] Friedman Y., Balaga O. & Linial M. Working together: combinatorial regulation by microRNAs. Advances in experimental medicine and biology 774, 317–337, doi: 10.1007/978-94-007-5590-1_16 (2013). 23377980

[b35] McDonald M. K. & Ajit S. K. In Progress in molecular biology and translational science Vol. Volume 131 (eds Price Theodore J. & Dussor Gregory) 215–249, (Academic Press, 2015). 25744675 10.1016/bs.pmbts.2014.11.015 PMC12076179

[b36] McDonald M. K. *et al.* Functional significance of macrophage-derived exosomes in inflammation and pain. PAIN^®^ 155, 1527–1539, doi: http://dx.doi.org/10.1016/j.pain.2014.04.029 (2014). 24792623 10.1016/j.pain.2014.04.029 PMC4106699

[b37] Squadrito Mario L. *et al.* Endogenous RNAs Modulate MicroRNA Sorting to Exosomes and Transfer to Acceptor Cells. Cell Reports 8, 1432–1446, doi: http://dx.doi.org/10.1016/j.celrep.2014.07.035 (2014). 25159140 10.1016/j.celrep.2014.07.035

[b38] Ramanathan S. & Ajit S. K. MicroRNA-Based Biomarkers in Pain. Advances in pharmacology (San Diego, Calif.) 75, 35–62, doi: 10.1016/bs.apha.2015.12.001 (2016). PMC12074672 26920008

[b39] Alexander G. M. *et al.* Plasma amino acids changes in complex regional pain syndrome. Pain research and treatment 2013, 742407, doi: 10.1155/2013/742407 (2013). 24303215 PMC3835366

[b40] Hsu S. D. *et al.* miRTarBase: a database curates experimentally validated microRNA-target interactions. Nucleic acids research 39, D163–169, doi: 10.1093/nar/gkq1107 (2011). 21071411 PMC3013699

[b41] Ma X., Becker Buscaglia L. E., Barker J. R. & Li Y. MicroRNAs in NF-kappaB signaling. Journal of molecular cell biology 3, 159–166, doi: 10.1093/jmcb/mjr007 (2011). 21502305 PMC3104013

[b42] Warde-Farley D. *et al.* The GeneMANIA prediction server: biological network integration for gene prioritization and predicting gene function. Nucleic acids research 38, W214–220, doi: 10.1093/nar/gkq537 (2010). 20576703 PMC2896186

