# Supplementary material for: Regulation of proinflammatory genes by the circulating microRNA hsa-miR-939
Source: Sci Rep. 2016 Aug 8;6:30976. doi: 10.1038/srep30976 (PMC4976376; doi:10.1038/srep30976)
Supplement: Supplementary Information [file srep30976-s1.pdf]

Regulation of proinflammatory genes by the circulating microRNA hsa-miR-939

Marguerite K. McDonald,[a,d*] Sujay Ramanathan,[a*] Andrew Touati,[a] Yiqian Zhou,[b] Rushi U. Thanawala,[a] Guillermo M. Alexander,[c] Ahmet Sacan,[b] and Seena K. Ajit[a¶]

[a]Pharmacology & Physiology, [c]Neurology, Drexel University College of Medicine, 245 North 15th Street, Philadelphia, PA 19102, USA

[b]School of Biomedical Engineering, Science & Health Systems, Drexel University, 3141 Chestnut Street, Philadelphia, PA 19104, USA

[d]Gene Therapy Program, Perelman School of Medicine, University of Pennsylvania, Suite 2000, Translational Research Laboratories (TRL), 125 S. 31st Street, Philadelphia, PA 19104-3403, USA

* These authors contributed equally

¶ Corresponding author

Contact information of the corresponding author

Seena Ajit PhD
Pharmacology & Physiology
Drexel University College of Medicine
245 North 15th Street, Mail Stop 488
Philadelphia, PA 19102
Tel.: +1 215 762 2218
Fax: +1 215 762 2299
E-mail: seena.ajit@drexelmed.edu

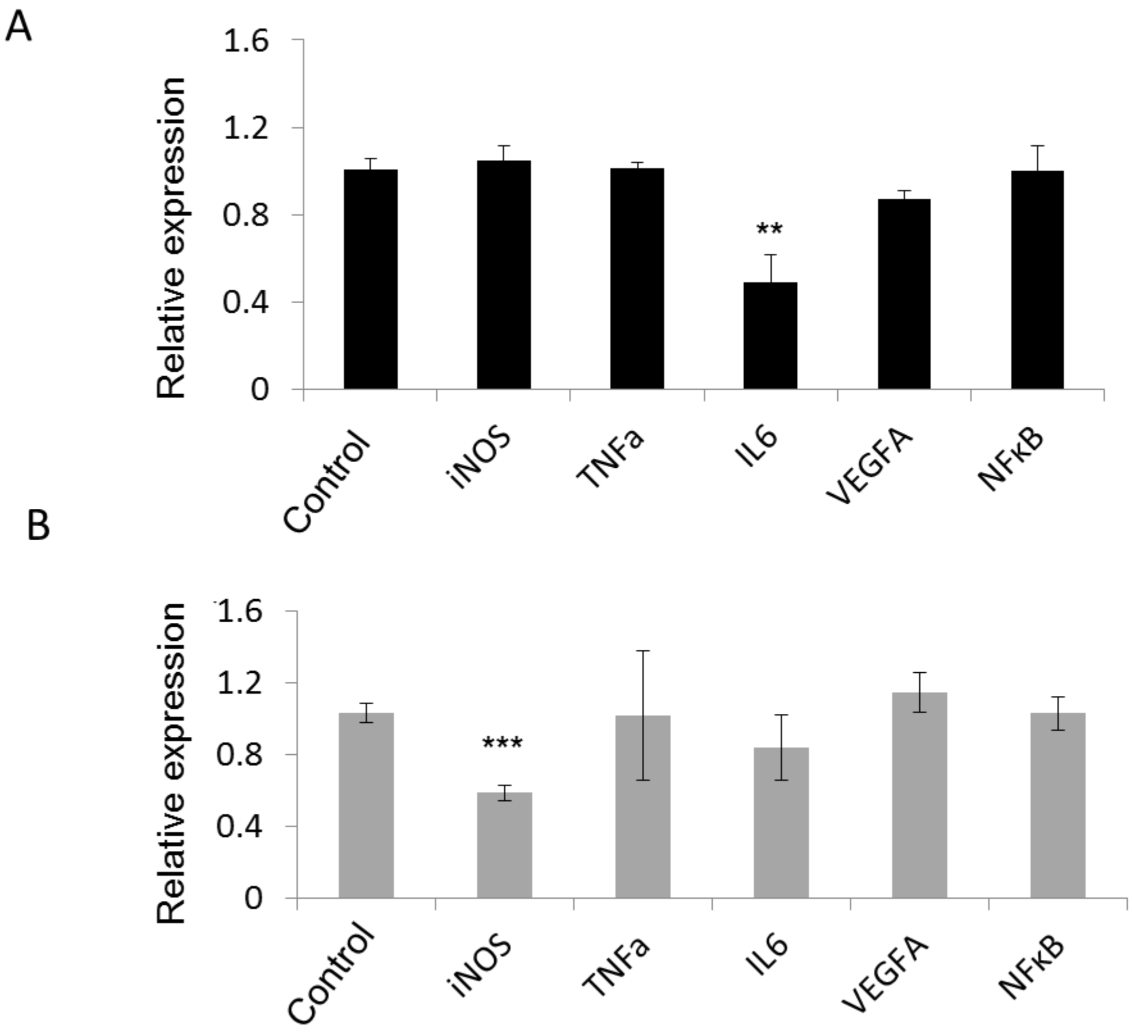

Supplementary Figure S1. Relative expressions of miR-939 target genes in THP-1 (A) and HUVEC (B) cells transfected with miR-939. Taqman analysis of endogenous levels of NOS2A (iNOS), TNFα, IL-6, VEGFA, and NFκB in THP-1 and HUVEC cells determined 24 hours after transfection with miR-939. *GAPDH* was used as a housekeeping gene. Significance was determined by one-way ANOVA $^{**}p < 0.01$, $^{***}p < 0.001$.

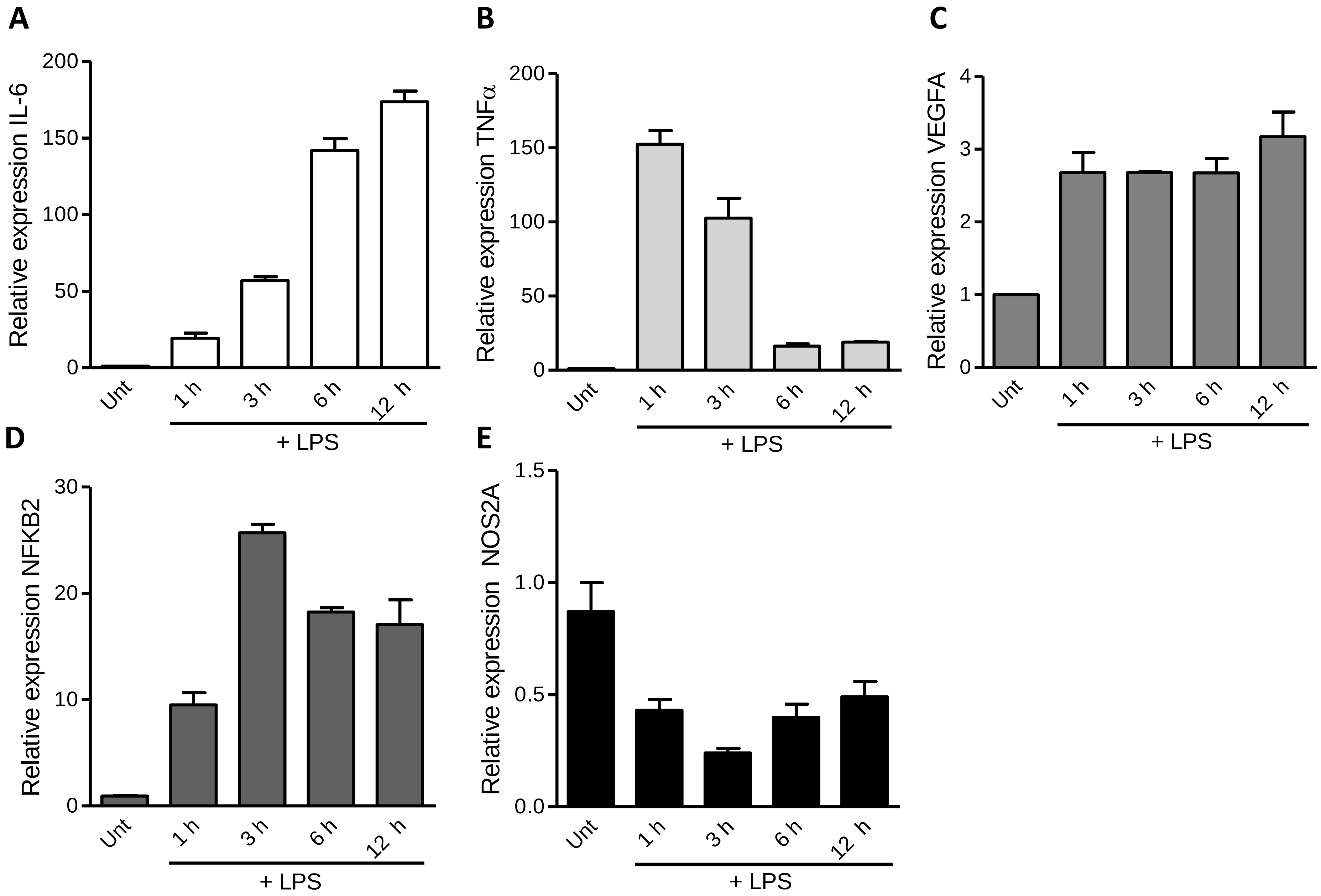

Supplementary Figure S2. Time course of LPS induced mRNA alterations for the miR-939 target genes in THP-1 cells. *GAPDH* was used as a housekeeping gene.

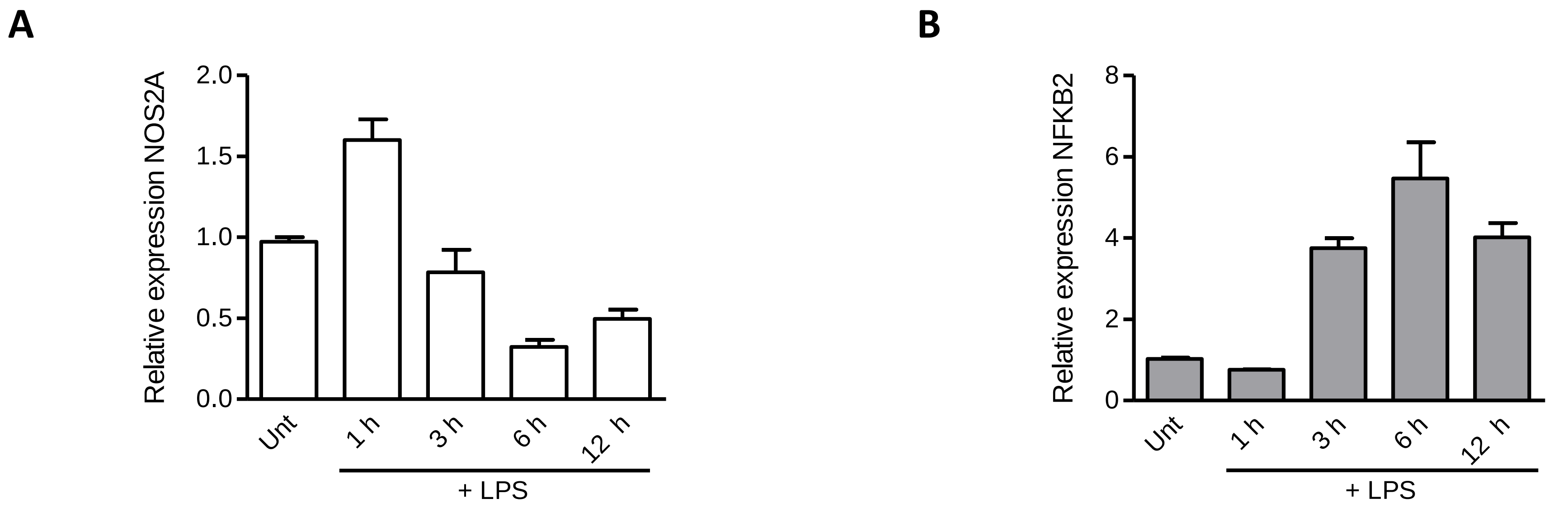

Supplementary Figure S3. Time course of LPS induced mRNA alterations for NOS2A and NFkB in HUVEC cells. *GAPDH* was used as a housekeeping gene.

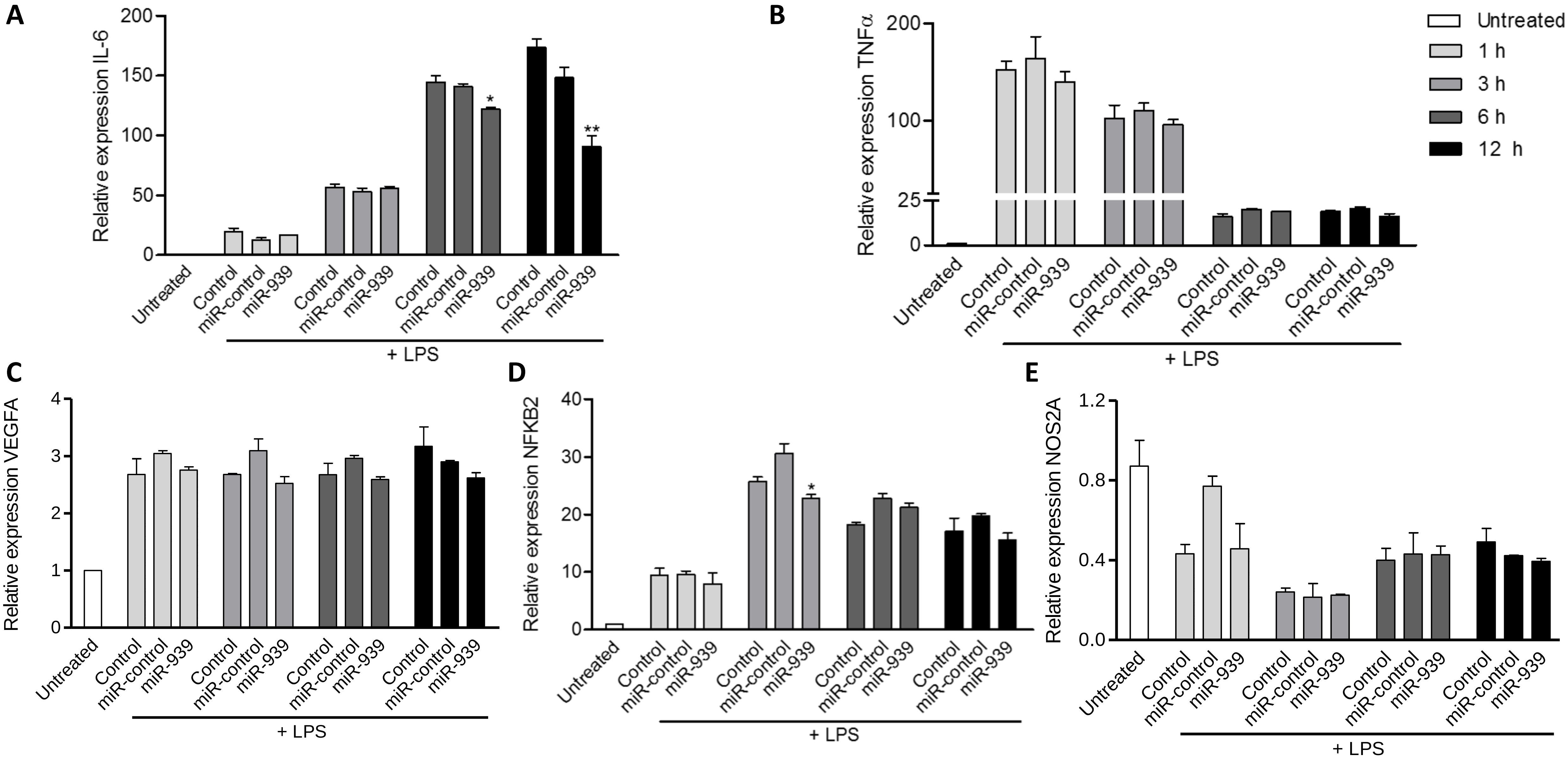

Supplementary Figure S4. Expression levels of miR-939 target genes IL-6, TNFα, VEGFA, NFκB2 and NOS2A in THP-1 cells transfected with miR-939 followed by LPS stimulation. Significance was determined by one-way ANOVA with Dunnet's post hoc test * $p < 0.05$, ** $p < 0.01$.

A

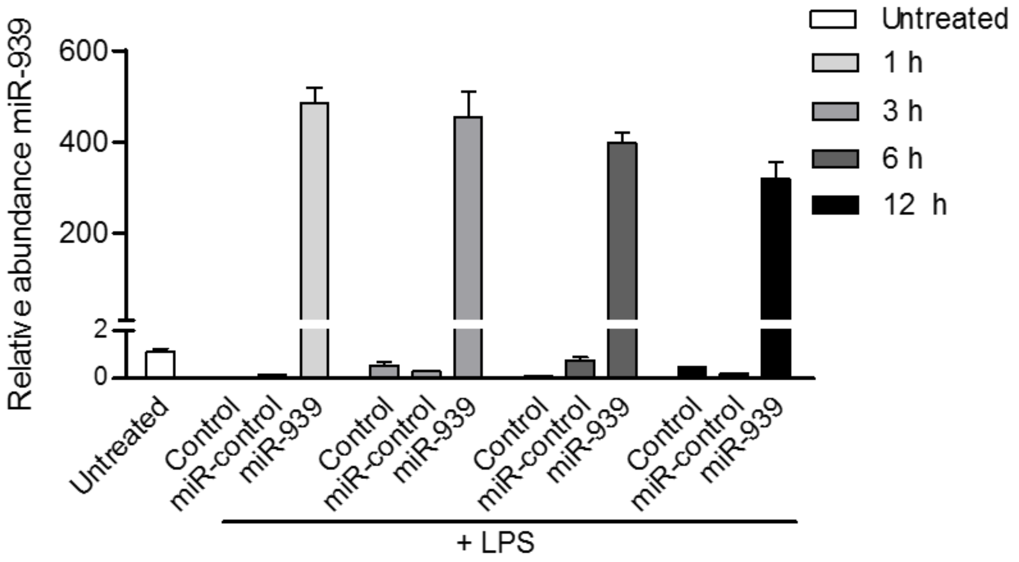

B

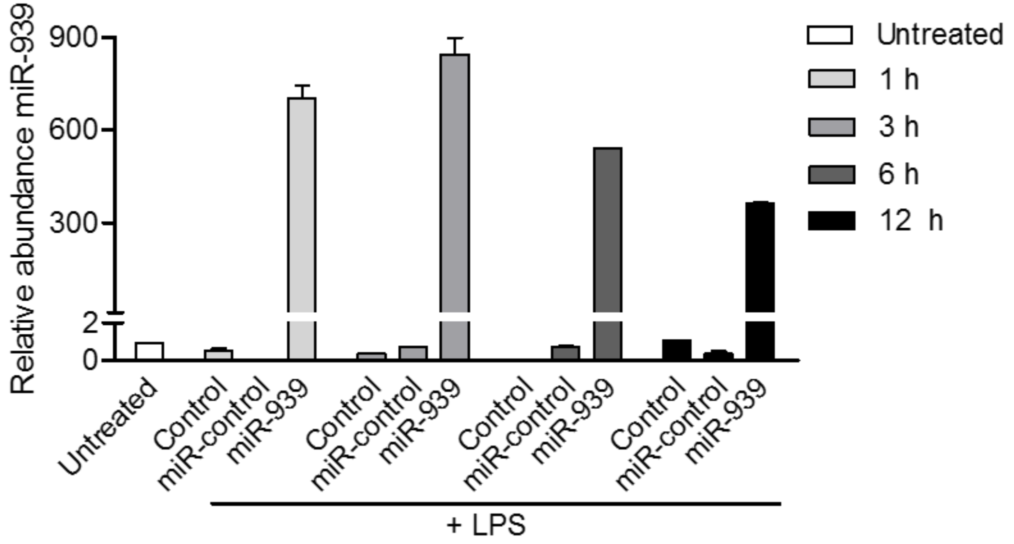

Supplementary Figure S5. Relative abundance of miR-939 in THP-1 (A) and HUVEC (B) cells transfected with miR-939 determined by qPCR. *U6* was used as the normalizer.
